# Improved Point-Cloud Segmentation for Plant Phenotyping Through Class-Dependent Sampling of Training Data to Battle Class Imbalance

**DOI:** 10.3389/fpls.2022.838190

**Published:** 2022-03-28

**Authors:** Frans P. Boogaard, Eldert J. van Henten, Gert Kootstra

**Affiliations:** ^1^Wageningen University & Research, Farm Technology Group, Wageningen, Netherlands; ^2^Rijk Zwaan Breeding, Fijnaart, Netherlands

**Keywords:** point-cloud segmentation, class imbalance, class-dependent sampling, plant phenotyping, deep learning

## Abstract

Plant scientists and breeders require high-quality phenotypic data. However, obtaining accurate manual measurements for large plant populations is often infeasible, due to the high labour requirement involved. This is especially the case for more complex plant traits, like the traits defining the plant architecture. Computer-vision methods can help in solving this bottleneck. The current work focusses on methods using 3D point cloud data to obtain phenotypic datasets of traits related to the plant architecture. A first step is the segmentation of the point clouds into plant organs. One of the issues in point-cloud segmentation is that not all plant parts are equally represented in the data and that the segmentation performance is typically lower for minority classes than for majority classes. To address this class-imbalance problem, we used a common practice to divide large point clouds into chunks that were independently segmented and recombined later. In our case, the chunks were created by selecting anchor points and combining those with points in their neighbourhood. As a baseline, the anchor points were selected in a class-independent way, representing the class distribution in the original data. Then, we propose a class-dependent sampling strategy to battle class imbalance. The difference in segmentation performance between the class-independent and the class-dependent training set was analysed first. Additionally, the effect of the number of points selected as the neighbourhood was investigated. Smaller neighbourhoods resulted in a higher level of class balance, but also in a loss of context that was contained in the points around the anchor point. The overall segmentation quality, measured as the mean intersection-over-union (IoU), increased from 0.94 to 0.96 when the class-dependent training set was used. The biggest class improvement was found for the “node,” for which the percentage of correctly segmented points increased by 46.0 percentage points. The results of the second experiment clearly showed that higher levels of class balance did not necessarily lead to better segmentation performance. Instead, the optimal neighbourhood size differed per class. In conclusion, it was demonstrated that our class-dependent sampling strategy led to an improved point-cloud segmentation method for plant phenotyping.

## Introduction

Plant scientists and breeders require high-quality phenotypic data. For example, phenotypic measurements of plant architecture-related traits are relevant to study the genotype–phenotype-environment relationship in the light of plant architecture. The plant architecture is the set of traits defining the three-dimensional (3D) organisation of the plant parts (Reinhardt and Kuhlemeier, 2002) and is an important indicator of plant stress (Suter and Widmer, 2013; Fahlgren et al., 2015; Paulus, 2019). Traits related to plant architecture are, for example, internode length, leaf angle, and leaf area. Measuring these traits manually with a high accuracy and a high temporal resolution is infeasible, due to the high labour requirement involved. Therefore, the availability of high-quality phenotypic data is often limited, especially for more complex traits, such as the traits related to plant architecture.

Computer-vision methods can increase the accuracy of the trait measurements and reduce the amount of manual labour involved. This allows to scale the phenotypic dataset in the number of time points per measurement as well as in the number of plants measured. Because of the complex 3D organization of the plant parts, we focussed on methods that are based on 3D point clouds using deep neural networks. In these methods, a first step that is often used, is to semantically segment the point cloud, such that for each point it is known to which plant part it belongs. The segmented point cloud can then be used as the basis for measuring plant traits. The aim of our work is to develop methods that can help in measuring traits related to the plant architecture to increase the availability of high-quality phenotypic data.

An issue that was identified in previous work on point-cloud segmentation is that point clouds of plants typically have a high level of class imbalance, with, for instance, an abundance of leaf points, but only few points belonging to classes such as “flower” or “node” (Boogaard et al., 2021; Turgut et al., 2022). This has consequences for semantic-segmentation methods, with the segmentation quality for the underrepresented classes lagging behind that of the overrepresented classes. For example, in the point clouds used by Boogaard et al. (2021), each of the classes “node,” “ovary” and “tendril” consisted of less than 1% of the points. The highest Intersection-over-Union (IoU, a metric to measure segmentation quality) reported for these classes was 0.23, while for the majority class “leaf,” the IoU was 0.99. In point clouds of roses (Turgut et al., 2022), the “stem” and “flower” were underrepresented as compared to the “leaf.” The highest observed IoU values for these underrepresented classes were 0.77 (“stem”) and 0.73 (flower), while the IoU for the overrepresented “leaf” was 0.95. In the current work, we propose a method to improve the segmentation of underrepresented classes, based on the level of class balance in the training data for the neural network.

### Class Imbalance—A Literature Review

The training procedure of a neural network consists of two main steps, creating the training data and training the network. In the first step, the available data needs to be manually labelled and transformed into a training, validation and test set. The training data are then used to train the network. The second step, training, depends on a set of hyper parameters like the neural network architecture, the loss function and the possibility of using pre-trained weights. In both steps, methods have been presented in literature to deal with class imbalance. We first discuss the approaches dealing with the training data and then the approaches dealing with the network and training procedure, in both cases focusing on 3D point clouds.

When preparing the training data, it is possible to repeat samples of underrepresented classes more often than samples of overrepresented classes. In the work of Lin and Nguyen (2020), randomly selected points of underrepresented classes were duplicated and randomly selected points of overrepresented classes were removed. This approach changes local point densities and structures, which could be prevented by oversampling entire objects instead of individual points. However, it is not straightforward to do this in semantic segmentation, since the recognition of an object also depends on the context in which it is presented. For example, the difference between a petiole and a stem is mainly visible because of the surrounding plant structure. One approach to do oversampling in a semantic-segmentation task was presented for the segmentation of LiDAR scans of urban areas. The point clouds in this work were divided into subsets, in this work referred to as *chunks*. A chunk was then duplicated if more than 70% of the points in that chunk belonged to underrepresented classes (Poliyapram et al., 2019). The possibility of applying data augmentation to duplicated chunks was added by Griffiths and Boehm (2019b), again in the context of 3D scanning and segmentation of an urban environment.

An alternative approach to reduce the level of class imbalance in the training set is to use synthetic data. An example was published by Griffiths and Boehm (2019a). They provided a large synthetic dataset of an urban environment in which the presence of small structures like poles and street furniture was increased, as compared to, for example, roads and pavements. In the plant domain, a synthetic dataset of sweet-pepper images including class and depth labels was generated by Barth et al. (2018) and in the work of Turgut et al. (2022), synthetic 3D rosebush models were used to train neural networks to segment plants from the ROSE-X dataset (Dutagaci et al., 2020). Such synthetic datasets could be modified to increase the focus on minority classes.

Also during training, class imbalance can be addressed. The training of a neural network is based on the loss, which is calculated from the difference between a predicted class and the ground truth. A common loss function for semantic segmentation is the cross-entropy loss, which gives equal weights to errors in each of the classes. The focus of a learning algorithm can be shifted towards minority classes by adding per-class weights to the loss function. These weights are then used to increase the loss for minority classes and decrease the loss for majority classes. This approach is known as weighted cross-entropy loss (Milioto et al., 2019). Focal loss is another variant of a weighted loss function in which the loss is inversely scaled by the probability that a point is of a certain class, which allows to obtain focus on minority classes (Lin et al., 2017).

Another way to deal with class imbalance is through pre-training of a neural network. Pre-training means that the network is first trained on a larger, often publicly available dataset, to learn common features. The dataset-specific features are then learnt in a second training procedure, based on the specific training set. This approach was adjusted to learn features of underrepresented classes first, before moving to the overrepresented classes, known as incremental transfer learning (Sander, 2020). The idea was to limit the dataset first to the underrepresented classes meaning that it is easier for the network to learn features of these classes. Then, the overrepresented classes are added, possibly in multiple steps. A strict stopping condition is required to prevent overfitting when the overrepresented classes have been added.

Although all the above methods alleviate the problem of class imbalance to some extent, new methods are needed to further improve the segmentation of underrepresented classes. In the current work, we focus on improving the level of class balance in the training set. The methods dealing with the network and training procedure are out of the scope of the current work.

### Contributions of the Paper

In the literature presented above, a common approach was to divide the point clouds into chunks that were then independently processed by the network. The main reason to create these chunks was that large point clouds cannot be processed at once for reasons of limited available memory storage and computational power. However, the chunks were either created based on fixed volumes, or by applying a sliding window that covered the entire point cloud. Instead of covering the entire point cloud, our aim was to show that the procedure to create the chunks could be designed such that the chunks were directly created with a focus on minority classes to improve the class balance in the training set. The main principle of our proposed method was based on two steps. First, anchor points were sampled from the point cloud and second, a set of points around each anchor point was selected as the chunk.

The focus of our method was to increase the level of class balance in the training set, based on the hypothesis that a higher level of class balance would lead to a better segmentation method. To test this, first, a reference training set was created in a class-independent way. In this class-independent training set, the class distribution of the sampled anchor points was proportional to the original class distribution. A second, class-dependent and training set was then generated, in which the class distribution of the sampled anchor points was inversely proportional to the original class distribution. In the first experiment, the added value of the class-dependent sampling approach was tested by using both training sets to train a neural network and comparing the performance on the segmentation task.

Although the anchor points were sampled from a specific class, the neighbourhood could contain points of different classes. So, even if an equal amount of anchor points was selected for each class, the neighbouring points of different classes reduced the level of class balance in the training set. By decreasing the number of points in the selected neighbourhood, this effect could be reduced. However, the neighbourhood of an anchor point does contain relevant information about the context of the anchor point. The second experiment, therefore, focussed on the effect of the number of points added as the neighbourhood on the level of class imbalance and the segmentation performance.

In this work, cucumber was used as a model crop. The neural network architecture used for the segmentation was PointNet++ (Qi et al., 2017). PointNet++ is one of the top performing neural network architectures for point cloud segmentation (Guo et al., 2020). In the plant domain, PointNet++ was, for example, used in roses (Turgut et al., 2022) and in cucumber (Boogaard et al., 2021). In both works, it was found that PointNet++ was a suitable basis for semantic segmentation in the plant domain as compared to other deep neural networks.

## Materials and Methods

In this section of the paper, we first present how the point-cloud data of the cucumber plants were obtained and labelled in section “Data Acquisition.” In section “Class Imbalance,” the concept of class imbalance is introduced. The method to create the training sets and how this method was used to battle class imbalance is explained in section “Using the Division Into Chunks to Battle Class Imbalance.” The training procedure is then presented in section “Training Procedure” and the testing procedure and evaluation criteria are introduced in section “Testing Procedure and Evaluation.”

### Data Acquisition

**Figure 1:**
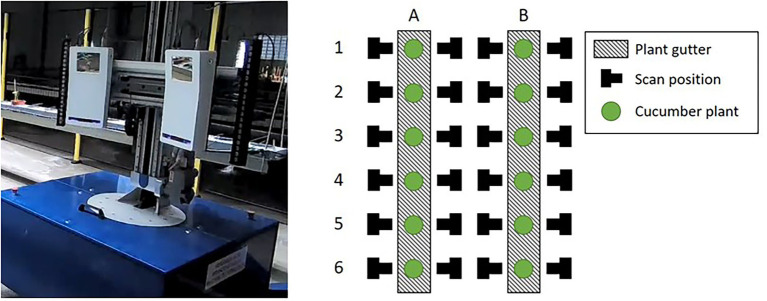
The mobile platform (left) including the Phenospex F500 Dual-scan system. The right image shows a schematic top-down overview of the plants in the climate chamber. The mobile platform automatically moved to the scan positions. At each position, the corresponding plant was scanned in a vertical direction by moving the scanners upwards (Boogaard et al., 2021).

**Figure 2:**
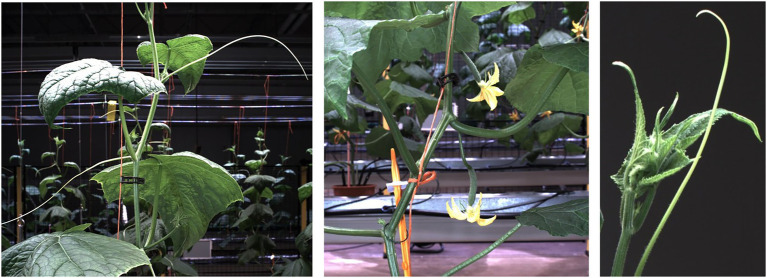
Impression of the different parts of the cucumber plants. The left image contains leaves, stem, petioles, tendrils and the supporting wire. In the middle image, two emerging fruits with flowers, in this research classified together as “ovary,” are visible. Also, some nodes are clearly visible in this image. The right image shows the growing point of the plant and another tendril.

The cucumber plants used for this research were of the variety Proloog RZ F1 (Rijk Zwaan, De Lier, Netherlands). Twelve plants were grown in a climate chamber on plant gutters. The plants were identified based on the location in the plant gutter as plant A1 up to B6, see Figure 1. An in-row distance between the plants of 1 m was used to prevent occlusion. The data-acquisition period started when the plants had 8 leaves and a plant length of 76 cm on average. After 11 days, at the end of the data-acquisition period, the average number of leaves was 12 and the average plant length was 195 cm. An impression of the plants is shown in Figure 2.

The point clouds used in this research were obtained using a Phenospex F500 Dual-scan^1^ system, based on laser-line triangulation. A driving WIWAM plant analyser^2^ (see Figure 1) was used to automatically position the scanners in front of each plant. The plants were then scanned in a vertical direction, from the plant gutter up to the growing point of the plant. Scans were made at both sides of the plant gutter. A schematic overview of the plants and the scanning positions is also given in Figure 1. The 24 scanning positions and the 11 days on which the plants were scanned resulted in a total dataset of 24 • 11 = 264 point clouds.

Next to the 3D spatial information, the Phenospex F500 Dual-scan system also provided colour information. So, the input data contained six features for each point in the point clouds: *x*, *y*, and *z* for the 3D position of the point and red, green and blue (*r*, *g*, and *b*) for the colour of the point.

**Figure 3:**
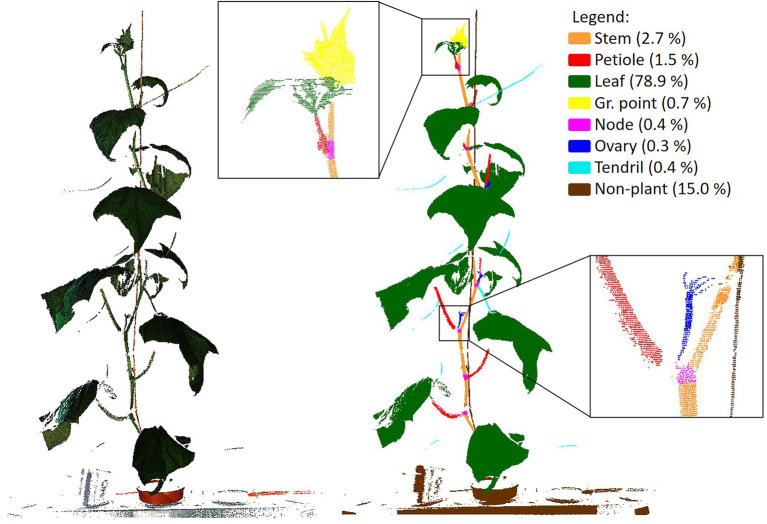
Example of an original, coloured, point cloud (left) and a manually segmented point cloud showing the 8 classes used in this research. In the legend, the fraction of points per class is given. The black squares show a zoomed in segment of the segmented point cloud.

To train PointNet++ and to be able to assess the quality of the trained network, a ground truth dataset was generated by manually segmenting all the 264 point clouds in the classes “stem,” “petiole,” “leaf,” “growing point,” “node,” “ovary,” “tendril” and “non-plant,” using the segment module of CloudCompare (CloudCompare, 2019). The class “non-plant” was used for the plant gutter, the pot, the plant label and the wire to which the plant was attached for support. An example of a coloured point cloud and a manually segmented point cloud is shown in Figure 3.

### Class Imbalance

In Figure 3, the percentage of points in each of the classes is added in the legend of the figure, clearly indicating the imbalance in the dataset. Most of the points (78.9%) were “leaf” points and also the “non-plant” class was relatively large (15.0%). The classes “stem” (2.7%) and “petiole” (1.5%) were a lot smaller, while each of the remaining classes (“growing point,” “node,” “ovary” and “tendril”) contained less than 1% of the points.

The level of class imbalance was quantified using the Shannon entropy, according to Eq. 1:


(1) $$h\left(d\right)=-\overset{C}{\underset{c=1}{\sum }}p_{c}^{d}•\log_{2}\left(p_{c}^{d}\right)$$

Where *h(d)* is the entropy in bits for dataset *d*, referring to either the manually labelled dataset, or one of the training sets created for the experiments done in this research. The number of classes is *C* and $p_{c}^{d}$ is the probability that a randomly chosen point belongs to class *c* in dataset *d*, which is equal to the fraction of points in dataset *d* belonging to class *c*. The upper limit for the entropy can be found for a completely homogeneous dataset, which is the case when all classes are equally present in the dataset. For the 8 classes labelled in our dataset, the maximum value for the entropy was $-\left(8•\frac{1}{8}•\log_{2}\left(\frac{1}{8}\right)\right)=3$ bits. If a dataset is dominated by one of the classes, the entropy decreases and approaches 0 bits. The entropy of the manually segmented dataset, based on the class distribution ass shown in Figure 3, was 1.1 bits.

### Using the Division Into Chunks to Battle Class Imbalance

The process in which the original point clouds were divided into chunks was used as a mechanism to battle class imbalance in this work. In our procedure, the chunks were created in two steps. First, for each class, a pre-defined number of anchor points was sampled from the point cloud. An anchor point for a certain class was a randomly selected point from the set of all points of that class. In the second step, the neighbouring points were added to each of the anchor points, based on a *k*-nearest-neighbour search. This search was performed on the entire point cloud, including points of different classes than the class of the anchor point. The selected neighbourhood was then saved as a training sample. The value of *k* was first set at 4,096, as this is a value often used in literature.

The first experiment to battle class imbalance focussed on the selection of anchor points from the point cloud. The aim was to decrease the level of class imbalance by changing the number of anchor points per class, based on two strategies. For the first strategy, the anchor points were selected in a class-independent manner to maintain the original class distribution as a reference. So, the number of anchor points per class was based on the original class distribution. For each class c, the number of anchor points *a_c_* was defined by Eq. 2, where *n_c_* is the number of points in class *c*, *n* is the total number of points in the point cloud and *a* is the number of anchor points per point cloud. The value for *a* was set at 100, as a balance between processing time and the amount of data used for training.


(2) $$a_{c}=\frac{n_{c}}{n}•a$$

The second training set was created using a class-dependent selection of anchor points. The hypothesis of this strategy was that a class-dependent selection of anchor points could reduce the level of class imbalance and improve the segmentation performance. For the class-dependent strategy, the number of anchor points per class was based on the inverse of the original distribution, according to Eq. 3. First, the inverse fraction of points in a class was calculated as 1 minus the fraction of points in that class. The inverse fraction for each class was then divided by the sum of the inverse fractions for all classes, in order to obtain the fraction of anchor points for this class in the training set. The obtained fraction was multiplied by the total number of anchor points per point cloud, *a*, to obtain the number of anchor points per class. As in the previous strategy, the value for *a* was set at 100.


(3) $$a_{c}=\frac{1-\frac{n_{c}}{n}}{\sum_{i=1}^{C}\left(1-\frac{n_{i}}{n}\right)}•a$$

**Figure 4:**
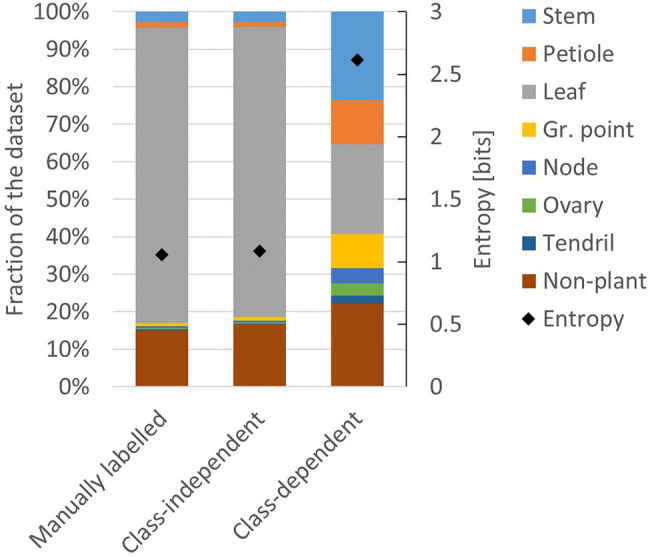
Composition of the manually labelled dataset, the class-independent training set and the class-dependent training set. The black diamonds show the level of entropy (secondary axis) according to Eq. 1.

The two resulting training sets are referred to as the class-independent and the class-dependent training set. The resulting class distribution in these two training sets is presented in Figure 4. For comparison, the class distribution of the manually labelled dataset is also shown. The entropy of the manually labelled data and the class-independent training set was both 1.1 bits, while the entropy of the class-dependent training set was 2.6 bits. This indicates that the class-dependent training set had a higher level of class balance.

**Figure 5:**
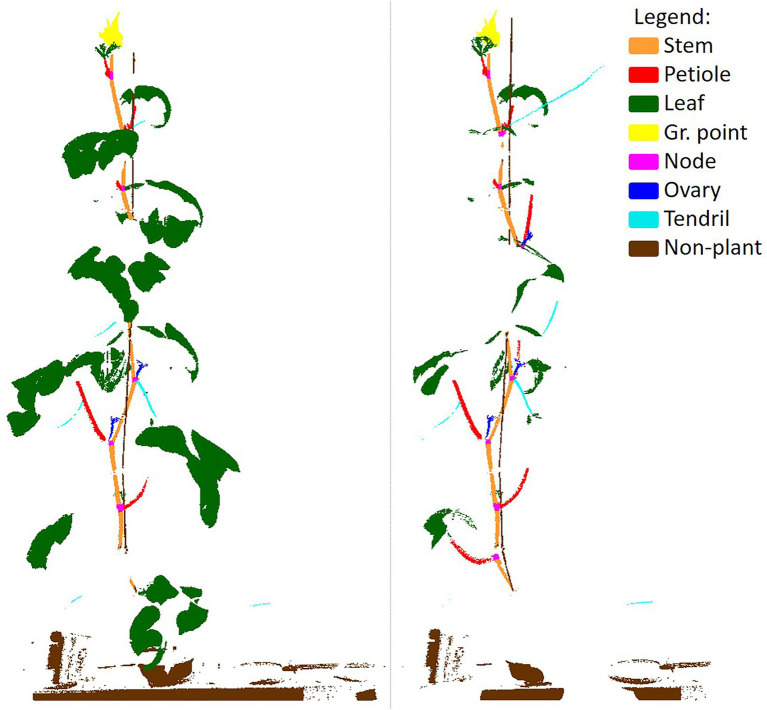
Example of the selected points for the class-independent (left) and the class-dependent (right) training set. The class-dependent training set was more balanced. Note that points could be included multiple times in overlapping chunks, which is not visible in this figure.

To provide more insight into how the two training sets differ from each other, the points that were selected for training for one point cloud are shown in Figure 5. The left image shows the selected points for the class-independent training set and the right image shows the selected points for the class-dependent training set. In the right image, the increased focus on the underrepresented classes (“node,” “ovary” and “tendril,” but also “stem” and “petiole”) and the decreased focus on the overrepresented classes (“leaf” and “non-plant”) is clearly visible.

Although the level of class balance in the class-dependent training set was increased, there was still quite some variation in the presence of classes. The smallest class (“tendril”) occupied 2% of the points, while the largest class (“leaf”) still occupied 24% of the points. The main reason was that the selected neighbourhood contained points of different classes than the class of the selected anchor point. The number of points in a chunk that were of a different class than the targeted anchor point depended on the physical size of the target class in the point cloud as well as the value of *k*. In fact, to obtain a completely homogeneous dataset, an equal amount of anchor points from each class could be selected and the chunk size could be set to 1, to prevent the inclusion of neighbouring points of different classes. However, this would also cause the complete loss of the context of the selected anchor point. Neighbouring points add information about local point densities and geometrical aspects like the curvature of the plant part. To provide an intuition about why this is important, consider that an individual point contains only six features: its x, y, and z location and the r, g and b colour. If only these six features would be known, especially in the current data, it is infeasible to know to what class a point belongs. The features of neighbouring points help to predict the correct class.

**Figure 6:**
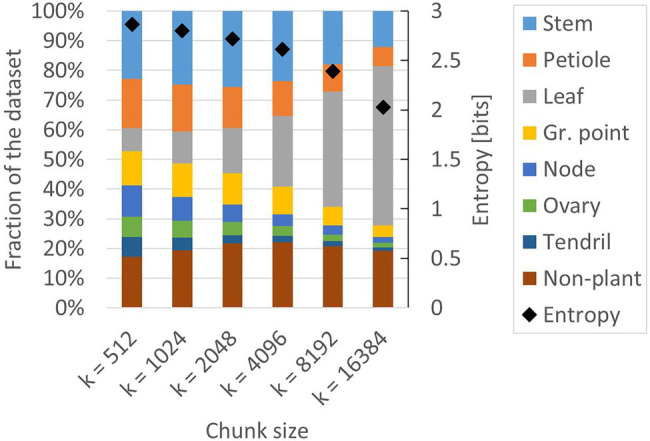
Composition of the six training sets created for experiment 2. All datasets are based on the class-dependent sampling strategy using six different values for *k*. The black diamonds show the level of entropy (secondary axis) according to Eq. 1.

This apparent tension between class balance and context was further investigated in a second experiment. We created additional training sets based on the class-dependent strategy, using *k* = 512, *k* = 1,024, *k* = 2,048, *k* = 4,096, *k* = 8,192 and *k* = 16,384. The number of anchor points per point cloud, *a*, remained 100 for each of these training sets. The training set for *k* = 4,096 was the same as in experiment 1. The composition and the entropy for the generated training sets are shown in Figure 6. Indeed, the entropy for the smaller chunk sizes was higher than the entropy for the larger chunk sizes, indicating that the training sets for smaller values of *k* have a lower level of class imbalance. The variation in presence between the classes was lowest at *k* = 512, ranging from 7% (“ovary” and “tendril”) to 23% (“stem”).

**Figure 7:**
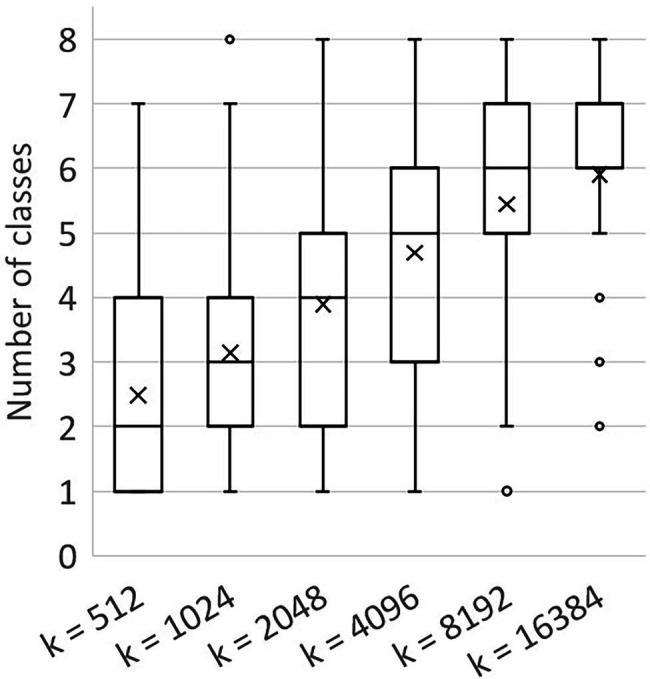
Boxplot showing the number of classes per chunk for different values of *k*, based on the training sets created using *a* = 100. The mean values are shown as “x.” The box shows the lower and upper quartile, the whiskers indicate the highest and lowest number of classes per chunk within 1.5 times the inter-quartile range. Values outside this range were considered outliers and are shown as a circle.

Besides context in the sense of the number of neighbourhood points (*k*), another way to look at the context of a point is to count the number of classes that were present in a chunk. The number of classes per chunk was analysed for the different values of *k*, as shown in Figure 7. The mean value for the number of classes per chunk increased for larger chunk sizes, indicating that a higher level of context was present in larger chunks.

In summary, a class-independent training set was generated for *k* = 4,096 and six class-dependent training sets were generated for *k* = 512, *k* = 1,024, *k* = 2,048, *k* = 4,096, *k* = 8,192 and *k* = 16,384. The training sets were used to train PointNet++, as presented in the next section.

### Training Procedure

The PointNet++ implementation published by Qi et al. (2018) was trained to segment the chunks of the point clouds into plant parts. The manually labelled data were first split into a training, validation and test set. To prevent that data from one plant were in more than one of these sets, this split was made on a plant level. As the result of a random selection procedure, all data of plant B6 were selected as the validation set and all data of plant A1 were selected as the test set (see Figure 1 for the plant IDs). In previous work using the same dataset (Boogaard et al., 2021), a cross-validation was done to quantify the variation in performance when different plants would be used for the training, validation and test split. For the population of plants considered, it was found that variation was low. Therefore, in the present research, no cross-validation was performed.

The aim of the network was to predict to which plant part each point belongs, generally called semantic segmentation. Besides features on a per point basis, the network calculated contextual features depending on the provided neighbourhood points. To obtain these features, a set-abstraction level was used, which consisted of three layers. In the sampling layer, a set of points was selected from the input chunk using a farthest point sampling algorithm. These points were grouped with points in their neighbourhood in the grouping layer. Finally, for each of the groups, a PointNet-based (Qi et al., 2016) layer was applied to learn the local point features. The set abstraction was then repeated to learn features on a larger scale. Because of the sampling and grouping, not all points were processed in this way. The feature vectors for unprocessed points were obtained by distance-based interpolation. No changes were made to the PointNet++ − architecture in our study, so for further details about the network, we refer to the work of Qi et al. (2016, 2018).

The training procedure used a sparse softmax cross-entropy loss. As mentioned in the introduction, the loss function could be changed to increase the loss for incorrect predictions of minority classes. Since we focussed on the effect of improved class balance in the training set, we did not change the loss function in the current research. After each training epoch, the loss on the validation set was monitored and training was stopped when this loss no longer decreased. The weights that corresponded to the lowest observed loss on the validation set were then used for further evaluation of the performance.

Since the training of a neural network is a stochastic process, the results can slightly differ per training run and therefore, the training was repeated five times for each configuration. The results, presented in section “Results,” are based on the average performance of these repetitions.

### Testing Procedure and Evaluation

As mentioned in section “Training Procedure,” all point clouds of plant A1 were used as the test set. This means that the data of this plant were not used in the training nor in the validation of the method, such that the performance of the method on this plant resembled the performance on new data. Similar to the training sets, the point clouds of the test set were split into chunks. Since the objective of the test set was to estimate the performance on new data, meaning that no manually assigned labels would be available, the class-independent sampling approach was used for the test set. As a consequence, the class distribution in the test set matched the class distribution in the original data.

Furthermore, to be able to segment the entire point clouds of the test set, it was necessary to make sure that all points were included in at least one of the chunks. Therefore, the number of chunks per point cloud, *a*, was not set on forehand for the test set. Instead, the selection of anchor points was repeated until all points of the point cloud had been added to at least one chunk. In this procedure, the anchor points were iteratively selected from the set of points that were not yet added to a chunk. To maintain local structures and point densities, the *k*-nearest neighbours were selected from the set of all points.

The trained network was then used to predict the segmentation of all chunks in the test set. The segmented chunks were merged to obtain the segmentation of the entire plant. Due to the way the chunks were created, points could be present in multiple chunks. If multiple predictions were present for a point, the predicted class in the merged point cloud was based on a majority-voting strategy including all individual predictions for that point. In case the voting ended in a tie, one of the classes in this tie was selected at random. The merged point clouds were used for the quantitative evaluation of the experiments. That is, the evaluation was done on the full point clouds of the plants.

The segmentation performance obtained in the experiments is reported as the intersection-over-union (IoU) between the manually labelled data and the predictions of the trained network for the test set. The IoU was based on a point-wise comparison between the manual and predicted point labels. A point was considered a true positive (TP) if the predicted label was equal to the manual label. If the predicted label was not equal to the manual label, a point was considered a false positive (FP) for the predicted class and a false negative (FN) for the manually assigned class. Based on the number of TP, FP and FN points, the IoU for class c was calculated according to Eq. 4:


(4) $${\mathrm{IoU}}_{c}=\frac{{\mathrm{TP}}_{c}}{{\mathrm{TP}}_{c}+{\mathrm{FP}}_{c}+{\mathrm{FN}}_{\mathrm{c[-]}}}$$

Besides the IoU values per class, the average IoU value is reported in two ways. First, the micro average of the IoU was calculated according to Eq. 5. This provided an indication of the IoU for the entire dataset on a point level. However, since the “leaf” was heavily overrepresented in the test set, this value was dominated by the IoU of the class “leaf.” Therefore, we also report the macro average, which is the average of the per-class IoU values. The macro average was calculated according to Eq. 6.


(5) $${\mathrm{IoU}}_{\mathrm{micro}}=\frac{\sum_{c=1}^{C}{\mathrm{TP}}_{c}}{\sum_{c=1}^{C}({\mathrm{TP}}_{c}+{\mathrm{FP}}_{c}+{\mathrm{FN}}_{c})[-]}$$

(6) $${\mathrm{IoU}}_{\mathrm{macro}}=\frac{\sum_{c=1}^{C}{\mathrm{IoU}}_{c}}{\mathrm{C[-]}}$$

where C is the total number of classes.

The precision (P) and recall (R) are also reported per class, based on Eqs. 7 and 8. The precision reports what proportion of points that was predicted as a certain class, actually belonged to that class. The recall reports what proportion of points that actually belonged to a certain class was also predicted as that class.


(7) $$P_{c}=\frac{{\mathrm{TP}}_{c}}{{\mathrm{TP}}_{c}+{\mathrm{FP}}_{\mathrm{c[-]}}}$$

(8) $$R_{c}=\frac{{\mathrm{TP}}_{c}}{{\mathrm{TP}}_{c}+{\mathrm{FN}}_{\mathrm{c[-]}}}$$

The significance of differences between reported IoU values was tested using a Wilcoxon signed-rank test. The outcome of these tests is reported as “n.s.” when the difference was not significant, as * when *p* < 0.05, as ** when *p* < 0.01 and finally as *** when *p* < 0.001.

## Results

In this section, the results of the two experiments are presented. First, in section “Selection of Anchor Points,” the segmentation performance for the class-independent and the class-dependent strategy used to select the anchor points is reported. In section “Number of Points Per Chunk,” the performance for the different chunk sizes is presented, based on the class-dependent sampling strategy. All results are based on the test set.

### Selection of Anchor Points

**Figure 8:**
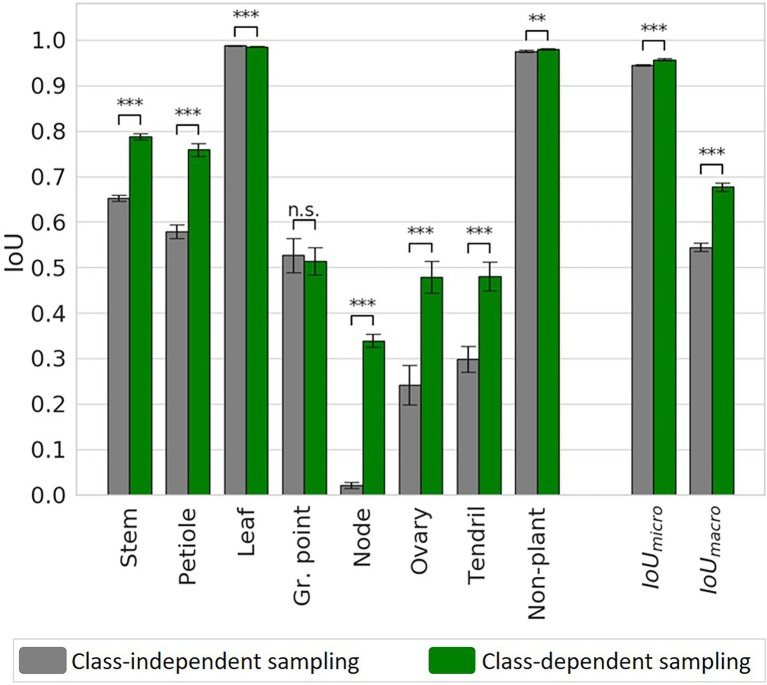
IoU for the class-independent and the class-dependent sampling strategy based on the test set. Bars indicate the mean IoU and the error bars give the 95% confidence interval on the mean. The archs indicate the significance of the differences between the observed IoU values.

The performance of the segmentation for the two sampling strategies, both with chunk size 4,096, is shown in Figure 8. When comparing the class-dependent sampling strategy to the original sampling strategy, for most classes and the average IoU values, a significant improvement of the segmentation quality was observed. Exceptions are the “growing point” (no significant difference) and the “leaf,” where a very small but significant decrease of the IoU was observed.

The biggest improvement was observed for the smallest classes, as they suffered most from the imbalance in the original dataset. The IoU increased from 0.02 to 0.34 for the “node,” from 0.24 to 0.48 for the “ovary” and from 0.30 to 0.48 for the “tendril.” The IoU values for the “stem” and “petiole” also increased, from 0.65 to 0.79 and from 0.58 to 0.76, respectively. Finally, the micro average of the IoU increased from 0.95 to 0.96 and the macro average of the IoU increased from 0.54 to 0.68, for the class-dependent sampling strategy.

The confusion matrices for the class-independent and class-dependent sampling strategy as well as the difference between the two are reported in Table 1. For the class-independent training set, the majority classes showed the highest percentage of correctly predicted points. For the classes “leaf” and “non-plant,” the percentage of correctly predicted points, shown on the diagonal, was 99.5 and 98.6%, respectively. Also the “stem,” “petiole” and “growing point” showed a high percentage of correctly predicted points, 86.7, 73.4 and 92.5%, respectively. The performance was lower for “ovary” (50.0%) and “tendril” (32.9%). For the class “node,” only 2.7% of the points was correctly classified by the network.

**Table 1:** Confusion matrices for the trained network using the class-independent selection of anchor points, the class-dependent selection of anchor points and the difference between the two.

Class-independent (percentage)		Predictions							
		Stem	Petiole	Leaf	Gr. point	Node	Ovary	Tendril	Non-plant
True labels	Stem	**86.7**	6.5	1.9	1.5	0.4	0.7	0.1	2.1
	Petiole	17.2	**73.4**	6.4	1.3	0.3	0.9	0.3	0.2
	Leaf	0.0	0.0	**99.5**	0.4	0.0	0.0	0.0	0.0
	Gr. Point	0.3	0.0	5.9	**92.5**	0.0	0.0	0.2	1.0
	Node	69.0	21.3	1.6	2.2	**2.7**	1.4	0.1	1.8
	Ovary	20.9	24.9	1.8	0.2	0.9	**50.0**	0.8	0.5
	Tendril	21.8	7.9	21.4	1.1	0.2	2.7	**32.9**	12.0
	Non-plant	0.7	0.0	0.5	0.1	0.0	0.0	0.0	**98.6**
**Class-dependent (percentage)**		**Predictions**							
		**Stem**	**Petiole**	**Leaf**	**Gr. point**	**Node**	**Ovary**	**Tendril**	**Non-plant**
True labels	Stem	**91.1**	1.7	0.5	0.9	3.2	0.5	0.2	1.9
	Petiole	3.2	**88.7**	3.2	0.7	2.9	0.6	0.6	0.1
	Leaf	0.1	0.2	**99.0**	0.5	0.0	0.0	0.1	0.1
	Gr. point	0.9	0.1	1.9	**96.1**	0.1	0.0	0.2	0.7
	Node	38.9	7.8	0.7	1.2	**48.7**	0.7	0.2	1.9
	Ovary	4.3	23.2	1.0	0.1	5.0	**65.4**	1.0	0.1
	Tendril	10.3	3.9	8.4	0.8	2.2	0.5	**63.8**	10.2
	Non-plant	0.5	0.0	0.2	0.1	0.0	0.0	0.1	**99.0**
**Difference (percentage point)**		**Predictions**							
		**Stem**	**Petiole**	**Leaf**	**Gr. point**	**Node**	**Ovary**	**Tendril**	**Non-plant**
True labels	Stem	**4.4**	−4.8	−1.5	−0.6	2.8	−0.2	0.1	−0.2
	Petiole	−14.0	**15.3**	−3.2	−0.6	2.6	−0.3	0.3	−0.1
	Leaf	0.1	0.1	**−0.5**	0.2	0.0	0.0	0.1	0.0
	Gr. point	0.6	0.1	−4.0	**3.6**	0.1	0.0	0.0	−0.3
	Node	−30.1	−13.5	−0.9	−1.0	**46.0**	−0.7	0.1	0.1
	Ovary	−16.6	−1.7	−0.8	−0.1	4.0	**15.4**	0.2	−0.3
	Tendril	−11.6	−4.0	−13.1	−0.3	2.0	−2.2	**30.8**	−1.8
	Non-plant	−0.2	0.0	−0.2	0.0	0.0	0.0	0.1	**0.4**

*Each row in the confusion matrices is based on the points that were labelled as that class by hand. The percentages indicate how these points were predicted by the network, meaning that values on the diagonal are correct predictions (marked in bold, also known as recall). Wrong predictions are highlighted in red and correct predictions are highlighted in green, brighter colours correspond to higher values*.

The values outside the diagonal show what percentage of points that was manually labelled as a certain class, was predicted as a different class. The most errors were made for “node” points, 69.0% of these points was predicted as “stem” and 21.3% was predicted as “petiole.” Also “ovary” and “tendril” were often incorrectly predicted as “stem” or “petiole.” Furthermore, for the class “tendril,” 21.4% of the points was predicted as “leaf.”

When training on the class-dependent training set, the percentage of correctly predicted points increased for most of the classes. The percentage of “node” points that was correctly classified by the network increased by 46.0 percentage points to 48.7%. This improvement was mainly due to fewer “node” points being predicted as “stem” and “petiole.” On the other hand, the number of “stem,” “petiole,” “ovary” and “tendril” points incorrectly predicted as “node” also increased slightly. The confusion between “stem,” “petiole” and “leaf” was strongly reduced. The percentage of “node,” “ovary” and “tendril” points that were predicted to be “stem” and “petiole” was also reduced, although still 38.9% of the “node” points was classified as “stem” and 23.2% of the “ovary” points was classified as “petiole.” Only for the class “leaf,” a decrease in the number of correctly classified points was observed of 0.5 percentage point. Still, 99.0% of the “leaf” points was correctly classified and the number of false positives for the “leaf” decreased.

The precision and recall for the network trained on the class-independent and class-dependent training set are reported in Table 2. For most classes, the precision and the recall were higher for the class-dependent training set. The recall for “leaf” and the precision for “growing point” were slightly lower for the class-dependent training set. For the class “tendril,” the precision for the class-dependent training set is 16 percentage points lower than for the class-independent training set. This was mostly due to “leaf” points incorrectly predicted as “tendril.”

**Table 2:** Precision (P) and Recall (R) per class, for the class-independent and the class-dependent selection of anchor points.

	Stem		Petiole		Leaf		Gr. point		Node		Ovary		Tendril		Non-plant	
	P	R	P	R	P	R	P	R	P	R	P	R	P	R	P	R
Class-independent	0.72	0.87	0.74	0.73	1.00	1.00	0.55	0.93	0.34	0.03	0.80	0.50	0.85	0.33	0.99	0.99
Class-dependent	0.85	0.91	0.83	0.89	1.00	0.99	0.51	0.96	0.55	0.49	0.89	0.65	0.69	0.64	0.99	0.99

*Per column, the highest value is highlighted in green and the lowest value is highlighted in red*.

### Number of Points Per Chunk

**Figure 9:**
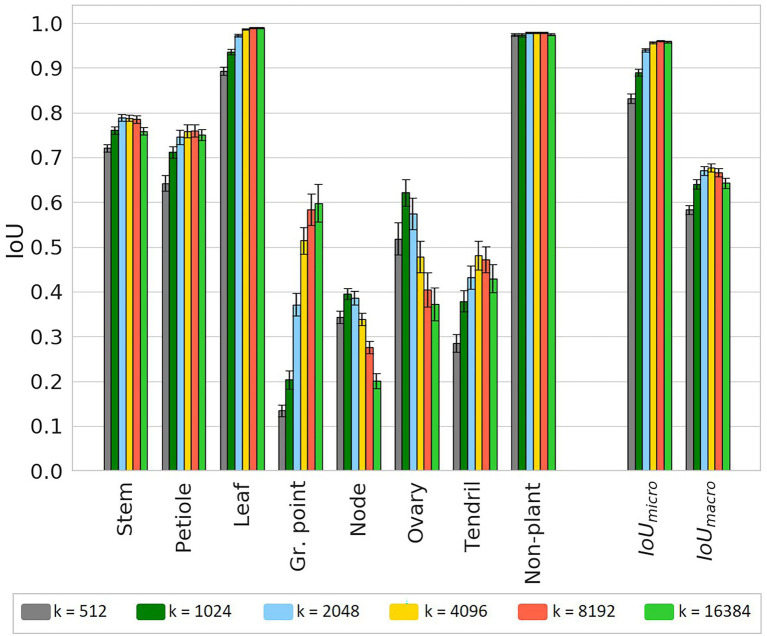
IoU for the test set using six different chunk sizes, based on the class-dependent sampling strategy. Bars indicate the mean IoU and the error bars give the 95% confidence interval on the mean. The significance of differences between the observed IoU values are reported separately in **Table A1** in Appendix A for readability.

The results of the experiment to test the effect of different chunk sizes on the segmentation performance are presented in this section. In Figure 9, the IoU values including the 95% confidence intervals on the mean are plotted. The significance of the differences for all classes are reported in Table A1 in Appendix A, based on a two-sided test.

For most classes, there seems to be an optimal chunk size at one of the intermediate values. For “stem,” the highest IoU was observed at *k* = 2,048; for “petiole,” the highest IoU was observed for *k* = 8,192. For these classes, the variation in IoU between *k* = 2,048, *k* = 4,096 and *k* = 8,192 was very small. For the classes “node” and “ovary,” the IoU was higher for smaller chunk sizes with a maximum at *k* = 1,024. However, for *k* = 512, the IoU was lower at a similar level as for *k* = 4,096. A similar pattern was found for the “tendril,” having the highest IoU at *k* = 4,096. The highest IoU for the class “non-plant” was found at *k* = 8,192, although this value was only significantly higher than the IoU for *k* = 16,384. For the classes “leaf” and “growing point,” the highest value was found at *k* = 16,384. However, it could be the case that, if even larger values for *k* were added to the experiment, also for these classes, the IoU would drop. On average, the results did show an optimal value at *k* = 8,192 for the micro average and at *k* = 4,096 for the macro average of the IoU.

To provide more insight into the precision-recall trade-off, the precision and recall for each class are reported in Table 3. For “stem” and “petiole,” both the precision and the recall decreased for smaller chunk sizes, caused by a higher fraction of FP and FN for these classes. The precision for the “leaf” stayed very high, meaning that if a point was classified as “leaf,” it almost certainly was correct. On the other hand, the recall for the “leaf” decreased for smaller chunk sizes, meaning that no longer all “leaf” points were retrieved for these chunk sizes. Based on visual inspection of the segmented point clouds, the main reason was identified as “leaf” points incorrectly classified as “growing point.” This also caused the drop in precision for the class “growing point.”

**Table 3:** Precision (P) and Recall (R) per class, for the six different chunk sizes.

Chunk size:	Stem		Petiole		Leaf		Gr. point		Node		Ovary		Tendril		Non-plant	
	P	R	P	R	P	R	P	R	P	R	P	R	P	R	P	R
512	0.82	0.85	0.74	0.82	1.00	0.90	0.07	0.96	0.49	0.53	0.74	0.80	0.32	0.77	0.99	0.99
1,024	0.84	0.89	0.80	0.85	1.00	0.94	0.12	0.96	0.56	0.58	0.90	0.79	0.46	0.76	0.98	0.99
2,048	0.86	0.91	0.83	0.87	1.00	0.98	0.29	0.97	0.58	0.57	0.89	0.75	0.54	0.71	0.99	0.99
4,096	0.85	0.91	0.83	0.89	1.00	0.99	0.51	0.96	0.55	0.49	0.89	0.65	0.69	0.64	0.99	0.99
8,192	0.85	0.91	0.84	0.88	1.00	0.99	0.61	0.94	0.50	0.39	0.87	0.60	0.77	0.59	0.99	0.99
16,384	0.82	0.92	0.84	0.87	1.00	1.00	0.64	0.89	0.46	0.29	0.88	0.55	0.78	0.54	0.99	0.99

*Values highlighted in green correspond to the higher values per column, while values highlighted in red correspond to the lower values per column*.

Looking at the classes “node” and “ovary,” the changes in precision and recall showed an optimum chunk size around 2,048 or 1,024 points. The optimum chunk size for “tendril” was 16,384 when aiming for precision, while it was 512 when aiming for recall. Finally, the precision and recall for the class “non-plant” were high and stable among the different chunk sizes.

## Discussion and Recommendations

### Data Set

The cucumber plants used in this research were grown in a climate chamber at an in-row distance of 1 meter. The distance between the plants prevented occlusion of plants by neighbouring plants. The main advantage of this experimental design was that each point cloud contained data of only one plant, which reduced the complexity of the segmentation task. For plant-phenotyping experiments, especially in data-acquisition systems based on a plant-to-sensor concept, it is a reasonable assumption that individual plants can be scanned. However, besides plant scientists and breeders, the presented methodology might be of interest for commercial growers to assess the current state of their crop. When measuring plants in a denser and more complex environment, resulting in plant–plant occlusions, for each plant part, it needs to be identified to which plant it belongs. This is an additional task, for which suitable methods need to be developed. The current dataset does not provide sufficient variation in complexity of the plants and their environment to study the effect of the level of complexity in the data on the segmentation performance. Extending the dataset in this direction would be valuable to further investigate practical applicability of the method for isolated plants as well as for high-density growing systems encountered in horticultural practice.

Although the measurement set-up resulted in high-resolution point clouds of individual plants, the point clouds were still not complete. That is, parts of the plant were missing in the point cloud, due to occlusion by other plant parts from the viewpoint of the scanner. Since PointNet++ relies on local geometrical features to segment the point clouds, it is likely that the missing data had a negative effect on the segmentation performance. A more in-depth investigation of this effect is recommended as future research. For this research, the level of completeness was the same for all experiments and it is not likely that it effected our results on dealing with class imbalance. We expect that the conclusions drawn at the end of the paper would not change if a more complete dataset was used.

The results presented in this paper clearly show that the class-dependent strategy outperformed the class-independent strategy. In this comparison, the class-independent strategy, with a chunk size of 4,096 points, was used as a baseline. Although it was shown in Figure 4 that the class distribution of the class-independent training set indeed matched the original class distribution, the training set was constructed using our sampling procedure instead of a sliding window approach. This means that also for the class-independent training set, not all points of the original point clouds were used for training, contrary to current practice. To further assess the validity of this training set as a baseline, the performance of the method trained on the class-independent training set was compared to the performance reported by Boogaard et al. (2021). In that work, a common sliding window approach was used and all points of the original point clouds were included in the chunks. The average difference between the IoU values of the classes was only 1 percentage point, and the micro and macro averages of the IoU were equal, indicating that the current class-independent training set provided a suitable baseline.

### Sampling of the Training Data

Seven training sets were created for the experiments, the class-independent training set for *k* = 4,096 and six class-dependent training sets for *k* = 512, *k* = 1,024, *k* = 2,048, *k* = 4,096, *k* = 8,192 and *k* = 16,384. The resulting training sets depended on three main parameters: the number of anchor points per class (*a_c_*), the number of anchor points per point cloud (*a*) and the method to define the neighbourhood of the anchor point, which was selected as the chunk. The effects of these parameters are discussed in the following three paragraphs.

First, in the current work, the number of anchor points per class was based on the inverse of the class distribution, instead of using an equal amount of anchor points per class. This resulted in a set of anchor points that contained many points from the minority classes such as “node” and “ovary,” and only few points from the majority classes, such as “leaf.” With a hypothetical setting of *k* = 1, this would result in a training set that would also be imbalanced but reversed to the original imbalance. This effect can be seen for *k* = 512 in Figure 6, where the original majority class “leaf” is now a minority class. Still, the training set based on *k* = 512 had the highest level of entropy, meaning that it was the most balanced training set. This is caused by the inclusion of neighbouring points, which were often from the original majority classes.

Second, the total number of anchor points per point cloud was set to 100 in the current work. The reason to keep this value constant was to obtain an equal amount of training samples, or chunks, for each of the values of *k*. However, instead of considering each chunk as a training sample, each individual point could be seen as a training sample. From that perspective, to keep the number of training samples constant, not the number of chunks, but the number of points in the training set (*a *∙ *k*) should be kept constant. In our case, the number of points in the training set increased for larger values of *k*. Although in general more training data lead to a better performance, the best performance for all classes except “leaf” was found at lower values of *k*, indicating the importance of class balance in the training set. It is recommended to further investigate the optimal number of anchor points per point cloud in relation to the chunk size. Besides saving on computation time, using less chunks also means that the effort needed for manually labelling the data can be reduced.

The third parameter was the algorithm used to select the neighbourhood of the anchor points. In this work, a nearest neighbour search was used, meaning that all selected points fitted within a sphere. In other applications, for example focussing on elongated objects, differently shaped neighbourhoods might be more suitable. Although different choices could have been made for the three parameters that were discussed, the conclusions in the light of the current research remain valid. It was clearly shown that the proposed class-dependent sampling method to create the training set did improve the segmentation quality.

### Evaluation

To evaluate the proposed method, the trained networks were used to segment the point clouds of a test set. To construct this test set, anchor points were added until all points of the point cloud were part of at least one chunk. This method can be relatively inefficient, as points can be included in multiple chunks. Based on the results of the majority-voting strategy to reconstruct the plants after segmentation, it turned out that having multiple predictions per point did not drastically improve the results, although this was not thoroughly investigated. Including fewer points in multiple chunks can reduce processing time. Therefore, an alternative approach to create the test set could be to apply a clustering algorithm that divides the input point cloud into clusters of the chosen chunk size. Unfortunately, most clustering algorithms do not guarantee a fixed number of members per cluster. A possible solution was presented by Yi and Moon (2014), who did propose a k-means based clustering method with a fixed number of cluster members. This and other alternatives to optimise the test set should be further investigated.

The evaluation of the segmented test set was based on the IoU, the precision and the recall. These are common evaluation criteria for semantic-segmentation methods and the results show that our proposed method outperformed the baseline with respect to these criteria. However, although the segmentation of the minority classes did improve, the performance for these classes is still lagging behind the performance for the majority classes. There is no general definition of when a segmentation method is good enough and therefore, it is difficult to assess the value of the proposed improvement. We recommend to evaluate the current state-of-the-art plant-segmentation methods not only in the light of computer-vision based criteria, like IoU, precision and recall, but also in the light of plant-based criteria like internode length, leaf angle and leaf area. This requires that during the data-acquisition phase of the phenotyping experiment, sufficient manual measurements of such plant traits need to be added to the dataset. Unfortunately, for the current data set, these measurements were not available.

### Final Recommendations

In this research, we focussed on the improvement of the segmentation quality that could be achieved using dedicated sampling of the training data. However, as mentioned in the introduction, the training procedure itself also provides opportunities to improve the segmentation performance on minority classes, for instance using data augmentation, weighted loss functions or synthetic data. Also increasing the amount of training data, possibly using multiple annotations per input sample, as suggested in Boogaard et al. (2021), could improve the segmentation. Evaluating these aspects goes beyond the scope of the current paper. We recommend to further explore these opportunities, quantifying the individual as well as the combined contributions to an improved segmentation method, in future work.

Finally, the class-dependent sampling method for creating the chunks was set-up in a generic way and is in principle not limited to cucumber plants. Specific settings, like the values for *k* and *a*, might be application or dataset specific. Therefore, the effect of these settings needs to be investigated to select a suitable value, similar to the way other hyper parameters of the network are set. The main procedure can then be tested on any point-cloud segmentation task. As imbalanced data are a typical problem in point cloud processing, also outside the plant domain, it would be interesting to test if the proposed method to create more balanced training sets also improves the segmentation performance in other applications and datasets.

## Conclusion

We have presented a method to battle class imbalance in the segmentation of point clouds of cucumber plants. As hypothesised, the results of the first experiment showed that the segmentation performance on the original, imbalanced and test data was significantly improved using a more balanced training set. As expected, the biggest improvements were found for the smallest classes. The percentage of correctly predicted “node” points increased by 46.0 percentage points, followed by the “tendril,” for which the percentage of correctly predicted points increased by 30.8 percentage points. Also the overall segmentation quality improved, with the micro average of the IoU increasing from 0.94 to 0.96 and the macro average of the IoU increasing from 0.54 to 0.68.

In the second experiment, the trade-off between the amount of context and the class balance was investigated. Six training sets were created for different values of the chunk size, *k*. This parameter defined the number of points sampled around each anchor point. Lower values for *k* tended to increase the level of class balance in the training set, while higher values added more context around the anchor point. The results showed that higher levels of class balance did not necessarily lead to better segmentation performance. The value for *k* showing the best segmentation results differed per class. Based on the average IoU values, the best segmentation results were obtained using *k* = 8,192 for the micro average or using *k* = 4,096 for the macro average.

In this paper, we have demonstrated that class-dependent sampling of the training data to improve the class balance in the training set led to an improved point-cloud segmentation method for plant phenotyping.

## Data Availability Statement

The raw data supporting the conclusions of this article will be made available by the authors, without undue reservation.

## Author Contributions

FB, EH, and GK contributed to the conception and design of the study. The data acquisition and development of the method were done by FB, guided by GK and EH. FB wrote the first draft of the manuscript. All authors contributed to the article and approved the submitted version.

## Conflict of Interest

The authors declare that the research was conducted in the absence of any commercial or financial relationships that could be construed as a potential conflict of interest.

## Publisher’s Note

All claims expressed in this article are solely those of the authors and do not necessarily represent those of their affiliated organizations, or those of the publisher, the editors and the reviewers. Any product that may be evaluated in this article, or claim that may be made by its manufacturer, is not guaranteed or endorsed by the publisher.

## Figures and Tables

**Table:** 

Point features	r, g, b	Spectral features: red, green, blue
	x, y, z	Geometric features (x, y, z location of point)
Division into chunks	*a*	Total number of anchor points per point cloud
	*a_c_*	Number of anchor points per class *c*, per point cloud
	*n*	Total number of points in point cloud
	*n_c_*	Total number of points in class *c*
	*k*	Number of points per chunk
Shannon entropy	*d*	Dataset, refers to the manually labelled dataset, or one of the training, validation and test sets
	*C*	Number of classes (*C* = 8 in the current research)
	*h(d)*	Shannon entropy for dataset *d*
	$p^{d}{}_{c}$	Probability that a randomly chosen point belongs to class c in dataset *d*
Evaluation	IoU_c_, IoU_micro_, IoU_macro_	Intersection-over-Union for class *c*, or as micro or macro average
	TP, FP, FN	True positive, False positive, False negative
	P, R	Precision, Recall

## Footnotes

^1^ https://phenospex.com/products/plant-phenotyping/planteye-f500-multispectral-3d-laser-scanner/

^2^ https://www.wiwam.be/projects/
