# Supplementary material for: Improved Point-Cloud Segmentation for Plant Phenotyping Through Class-Dependent Sampling of Training Data to Battle Class Imbalance
Source: Front Plant Sci. 2022 Mar 28;13:838190. doi: 10.3389/fpls.2022.838190 (PMC8996061; doi:10.3389/fpls.2022.838190)
Supplement: Supplementary file 1 [file Table_4.docx]

Appendix A

*Table A1 – Significance table, showing if the difference between IoU-values observed for different chunk sizes was significant, using a two-sided test. Which of the chunk sizes had a higher IoU can be seen in Figure 9. The table is symmetrical, for readability only the values below the diagonal are printed.*

|  | Chunk size | 512 | 1024 | 2048 | 4096 | 8192 |
| --- | --- | --- | --- | --- | --- | --- |
| Stem | 1024 | *** |  |  |  |  |
|  | 2048 | *** | *** |  |  |  |
|  | 4096 | *** | *** | n.s. |  |  |
|  | 8192 | *** | *** | n.s. | n.s. |  |
|  | 16384 | *** | n.s. | *** | *** | *** |
| Petiole | 1024 | *** |  |  |  |  |
|  | 2048 | *** | *** |  |  |  |
|  | 4096 | *** | *** | n.s. |  |  |
|  | 8192 | *** | *** | n.s. | n.s. |  |
|  | 16384 | *** | *** | n.s. | n.s. | n.s. |
| Leaf | 1024 | *** |  |  |  |  |
|  | 2048 | *** | *** |  |  |  |
|  | 4096 | *** | *** | *** |  |  |
|  | 8192 | *** | *** | *** | *** |  |
|  | 16384 | *** | *** | *** | *** | n.s. |
| Gr. point | 1024 | *** |  |  |  |  |
|  | 2048 | *** | *** |  |  |  |
|  | 4096 | *** | *** | *** |  |  |
|  | 8192 | *** | *** | *** | *** |  |
|  | 16384 | *** | *** | *** | *** | n.s. |
| Node | 1024 | *** |  |  |  |  |
|  | 2048 | *** | n.s. |  |  |  |
|  | 4096 | n.s. | *** | *** |  |  |
|  | 8192 | *** | *** | *** | *** |  |
|  | 16384 | *** | *** | *** | *** | *** |
| Ovary | 1024 | *** |  |  |  |  |
|  | 2048 | *** | ** |  |  |  |
|  | 4096 | ** | *** | *** |  |  |
|  | 8192 | *** | *** | *** | *** |  |
|  | 16384 | *** | *** | *** | *** | * |
| Tendril | 1024 | *** |  |  |  |  |
|  | 2048 | *** | *** |  |  |  |
|  | 4096 | *** | *** | *** |  |  |
|  | 8192 | *** | *** | *** | n.s. |  |
|  | 16384 | *** | *** | n.s. | *** | *** |
| Non-plant | 1024 | n.s. |  |  |  |  |
|  | 2048 | *** | ** |  |  |  |
|  | 4096 | * | n.s. | n.s. |  |  |
|  | 8192 | n.s. | n.s. | n.s. | n.s. |  |
|  | 16384 | n.s. | n.s. | *** | *** | *** |
|  |  |  |  |  |  |  |
| IoU_micro_ | 1024 | *** |  |  |  |  |
|  | 2048 | *** | *** |  |  |  |
|  | 4096 | *** | *** | *** |  |  |
|  | 8192 | *** | *** | *** | *** |  |
|  | 16384 | *** | *** | *** | n.s. | *** |
| IoU_macro_ | 1024 | *** |  |  |  |  |
|  | 2048 | *** | *** |  |  |  |
|  | 4096 | *** | *** | n.s. |  |  |
|  | 8192 | *** | *** | n.s. | ** |  |
|  | 16384 | *** | n.s. | *** | *** | *** |
