# Supplementary figures and images for: Improved Point-Cloud Segmentation for Plant Phenotyping Through Class-Dependent Sampling of Training Data to Battle Class Imbalance
Source: Front Plant Sci. 2022 Mar 28;13:838190. doi: 10.3389/fpls.2022.838190 (PMC8996061; doi:10.3389/fpls.2022.838190)

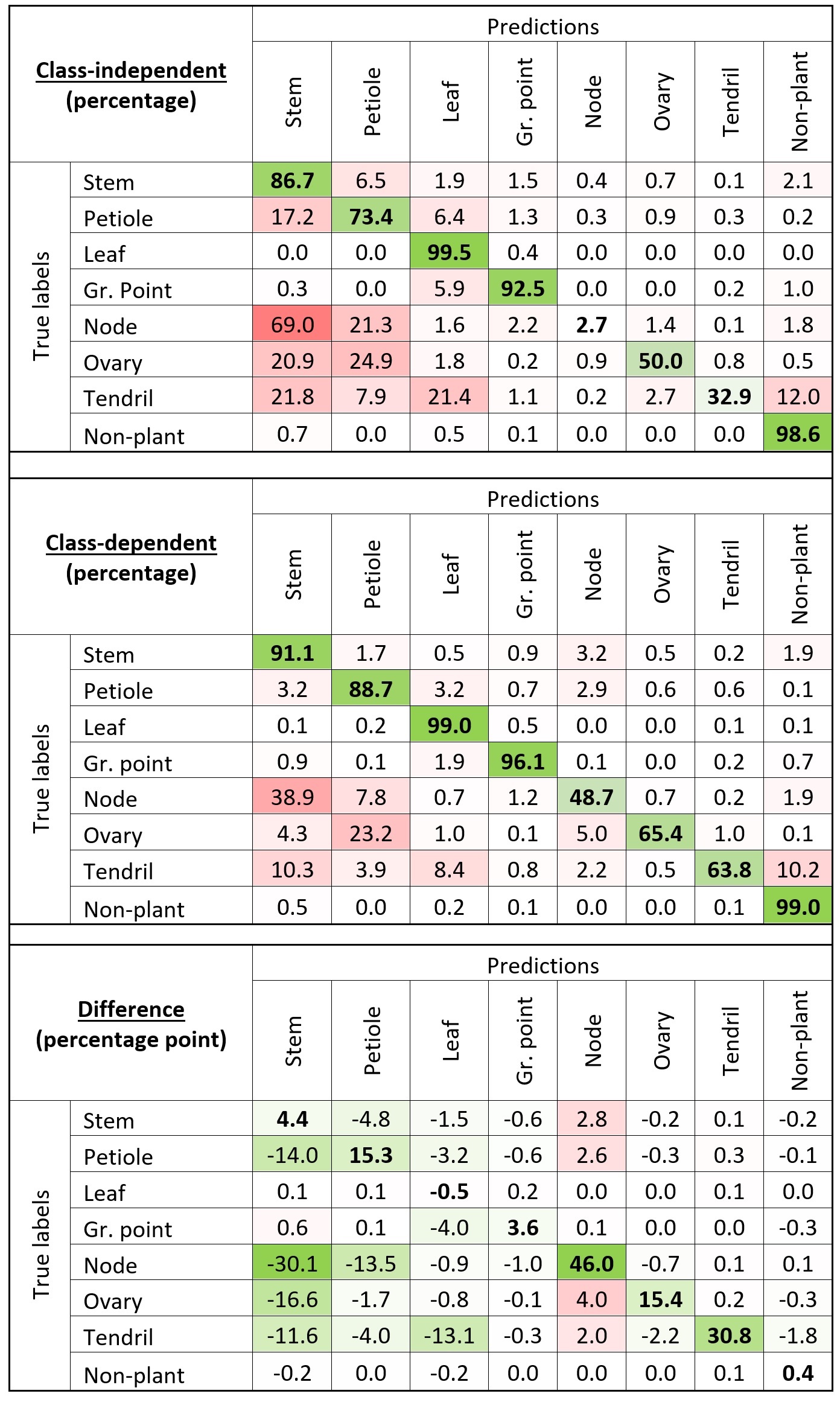

Supplement: Supplementary file 2 [file Image_1.jpg]

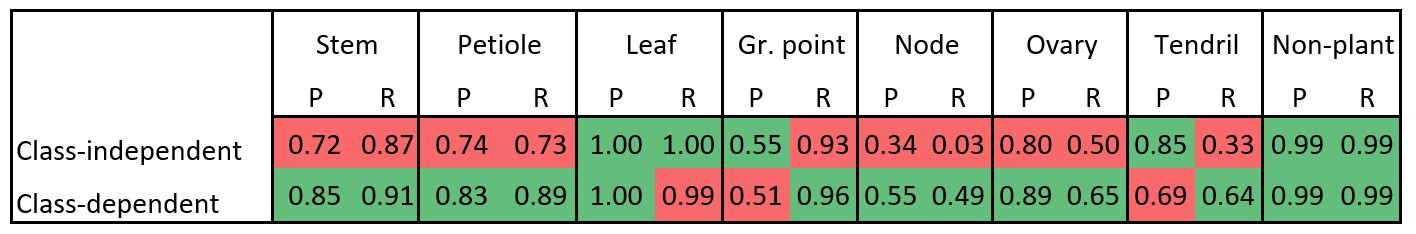

Supplement: Supplementary file 3 [file Image_2.jpg]

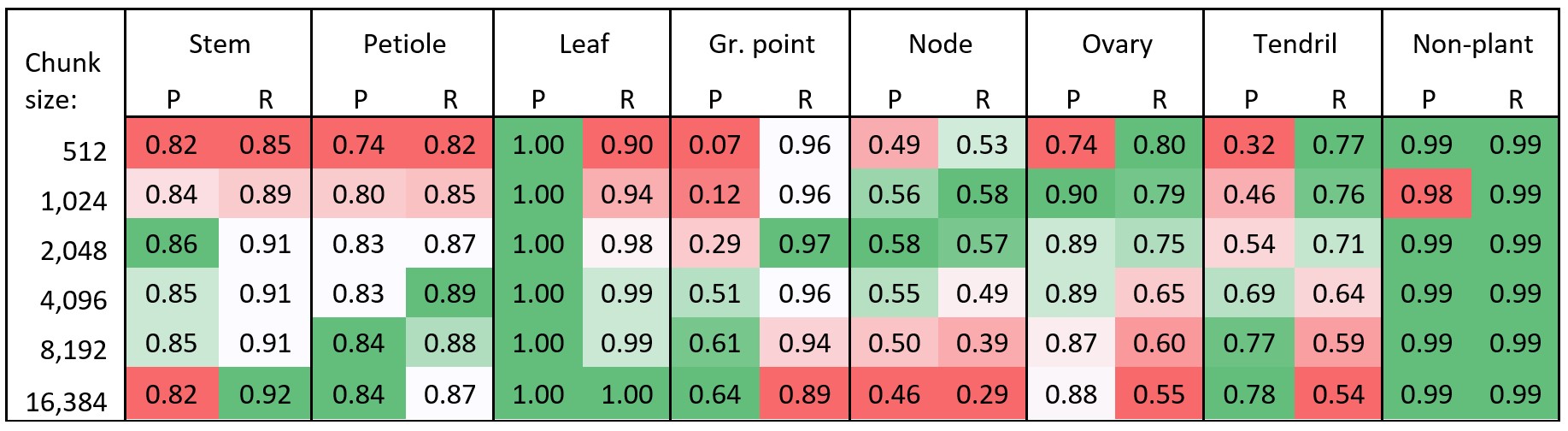

Supplement: Supplementary file 4 [file Image_3.jpg]
